# Prenatal Water Fluoride Exposure and Children’s Behavioral Problems

**DOI:** 10.1001/jamanetworkopen.2026.29389

**Published:** 2026-09-03

**Authors:** Alexis Hernandez, Anne E. Nigra, Katrina R. Simon, Tessa R. Bloomquist, Tushara Rajeev, Amy Margolis, Amii M. Kress, Mohamad Burjak, Lauren C. Shuffrey, Anahid Akbaryan, Xiaoye Xu, Chang Liu, Stephanie Eick, Zhu Wang, Judy L. Aschner, Hyeong-Moo Shin, Margaret Karagas, Rebecca C. Fry, Tiffany Sanchez, Theresa Bastain, Santiago Morales

**Affiliations:** 1 Department of Psychology, University of Southern California, Los Angeles; 2 Department of Environmental Health Sciences, Columbia University Mailman School of Public Health, New York, New York; 3 Department of Psychiatry and Behavioral Health, The Ohio State University, Columbus; 4 Clinical and Translational Science Institute, The Ohio State University, Columbus; 5 Department of Epidemiology, John Hopkins Bloomberg School of Public Health, Baltimore, Maryland; 6 Department of Child and Adolescent Psychiatry, NYU Grossman School of Medicine, New York, New York; 7 Psychology Department, SUNY Cortland, Cortland, New York; 8 Department of Psychology, Washington State University, Pullman; 9 Gangarosa Department of Environmental Health, Emory University Rollins School of Public Health, Atlanta, Georgia; 10 Department of Preventive Medicine, The University of Tennessee Health Science Center, Memphis; 11 Department of Pediatrics, Hackensack Meridian School of Medicine, Nutley, New Jersey; 12 Center for Discovery and Innovation, Hackensack Meridian School of Medicine, Nutley, New Jersey; 13 Department of Pediatrics, Albert Einstein College of Medicine, Bronx, New York; 14 Department of Obstetrics & Gynecology and Women’s Health, Albert Einstein College of Medicine, Bronx, New York; 15 Department of Environmental Science, Baylor University, Waco, Texas; 16 Department of Epidemiology, Geisel School of Medicine at Dartmouth College, Lebanon, New Hampshire; 17 Department of Environmental Sciences and Engineering, University of North Carolina at Chapel Hill; 18 Department of Epidemiology, Colorado School of Public Health, Aurora; 19 School of Public Health, Washington University, St Louis, Missouri

## Abstract

**Importance:**

Although fluoride exposure at high levels during the perinatal period has been associated with socioemotional problems among children, there is limited evidence regarding potential associations at lower levels of fluoride exposure, particularly in the US.

**Objective:**

To examine the association between prenatal exposure to public drinking water fluoride levels and children’s socioemotional problems in a pooled US pediatric consortium.

**Design, Setting, and Participants:**

This cohort study used observational pregnancy cohort data from January 1, 2005, to December 31, 2019, on children aged 1.5 to 17 years from the Environmental Influences on Child Health Outcomes (ECHO) Cohort, which took place at 20 prenatal pediatric cohort sites across 34 US states. Statistical analysis was conducted from December 2024 to December 2025.

**Exposure:**

Area-level, time-weighted, mean fluoride concentrations in public water were estimated based on residential addresses during pregnancy.

**Main Outcomes and Measures:**

Children’s socioemotional problems were assessed using caregiver reports of internalizing and externalizing problems via the Child Behavior Checklist. Generalized estimating equation models evaluated the adjusted mean difference in T scores (1) across the range of exposure values in cubic spline models, (2) per 500.0-µg/L higher fluoride level in linear models, (3) across quartiles of exposure, and (4) at the US Public Health Services recommended cutoff (≤700.0 µg/L; 3998 less than the cutoff).

**Results:**

The cohort comprised 5520 children (mean [SD] age, 5.8 [3.0] years; 2849 boys [51.6%]). The mean (SD) prenatal time-weighted water fluoride level was 407.9 (361.0) µg/L (range, <1.4-3272.5 µg/L). The adjusted mean difference in emotional and behavioral problem T scores per 500.0-μg/L higher exposure to prenatal public water fluoride was −0.15 (95% CI, −0.85 to 0.56) for internalizing problems and 0.06 (95% CI, −0.44 to 0.55) for externalizing problems, consistently showing no associations at the continuous level. However, some positive associations were observed only when using change point models for externalizing problems (adjusted mean difference, 1.59 [95% CI, 0.72-2.47] per 500.0-µg/L increase above the inflection point), and for some subgroups.

**Conclusions and Relevance:**

In this cohort study, prenatal exposure to fluoride levels in regulated public water was not associated with internalizing and externalizing problems among offspring. In change point models, positive associations were found for externalizing problems. The results contribute to growing evidence that could inform future policy decisions regarding fluoride in the US public water systems. Additional studies are needed to further investigate this association, especially examining other sources of fluoride that may have an association with children’s emotional and behavioral problems.

## Key Points

**Question:**

What is the association between prenatal exposure to public drinking water fluoride levels in the US and children’s emotional and behavioral problems?

**Findings:**

In this cohort study of 5520 US children, exposure to prenatal fluoride levels in regulated public water was not associated with internalizing and externalizing problems.

**Meaning:**

Results from the observational study add to growing evidence that could inform future policy decisions regarding fluoride in the US public water systems.

## Teaser

This cohort study examines the association between prenatal exposure to public drinking water fluoride levels and children’s socioemotional problems in the Environmental Influences on Child Health Outcomes Cohort.

## Introduction

Fluoride is state and locally regulated within the US public drinking system and used as a prevention tool for tooth decay.^1,2^ The recommended level of fluoride in the water by the US Public Health Services (USPHS) as of 2015 is 700.0 µg/L, selected to balance both dental benefits and avoid harms at the highest exposure levels.^2^ Ecological studies found that very high levels of fluoride exposure (>4000.0 µg/L in water) were associated with skeletal fluorosis, kidney and thyroid dysfunction, and negative effects on the central nervous system.^2,3^ There has been increased concern about the association of water fluoridation with children’s socioemotional problems, especially during the prenatal period, a window of susceptibility for systems involved in emotional and behavioral development.^4^ Exposure to fluoride in utero can be detrimental to the fetus as fluoride can pass through the placenta and disrupt normal cellular activity, possibly affecting central nervous system growth.^5,6^ However, few studies have tested these associations, specifically for socioemotional risk in US populations.

The few studies examining the associations between prenatal fluoride exposure and children’s emotional and behavioral problems have found that higher levels of prenatal fluoride exposure are associated with higher overall neurobehavioral problems, internalizing symptoms, and risk for attention-deficit/hyperactivity disorder.^4,7^ However, some of these studies used an ecological design (eg, state based or regional) or biospecimens (eg, maternal urine) rather than area-level exposure (eg, census tract level) via water. Water fluoride exposure has been studied in other countries in relation to children’s development,^8^ but it has not been well examined in US populations.^4^ Moreover, previous studies have focused mainly on postnatal fluoride exposure.^9,10^ Therefore, there is a need to understand how fluoride exposure via US public drinking water, especially during pregnancy via area-level exposure, is associated with child behavioral and emotional problems.

Previous studies in the US, including a study that partially overlaps with this sample,^4^ also have relied on biomarkers as their measure of postnatal exposure. Biomarkers integrate all exposure sources (eg, diet), making it challenging to isolate the specific contribution of water fluoride.^11,12^ This lack of source specificity can complicate the development of targeted interventions and regulatory insights. Therefore, examining the associations of drinking water fluoride exposure has a more direct interpretation and actionable results with regard to one of the major sources of fluoride exposure in the US population.^13^ Positive associations between fluoride concentrations in urine and drinking water (*r* = 0.79) have been reported across several populations,^14^ highlighting water as a major source of fluoride intake and its validity as a measure of exposure.

The present study evaluated the association between prenatal public drinking water fluoride exposure and child indicators of behavioral and emotional problems in the Environmental Influences on Child Health Outcomes (ECHO) Cohort.^15^ We hypothesized that we would observe positive associations between prenatal water fluoride exposure and children’s internalizing and externalizing problem T scores, consistent with previous studies examining prenatal fluoride exposure and children’s emotional and behavioral problems.^4,7^

## Methods

The ECHO Cohort (cycle 1) consisted of 69 ongoing pregnancy and pediatric cohort sites across the US and Puerto Rico.^16^ Protocols were approved by the study site’s local institutional board or ECHO central institutional review board. Written informed consent and parental permission were obtained before data collection. Children were eligible for the study if parents were recruited during pregnancy, reported their residential address, and resided in a census tract with available public water fluoride data for January 1, 2005, to December 31, 2019. In addition, we excluded participants if they had missing outcomes, came from an outcome-enriched cohort (eg, high familial likelihood of autism) or randomized clinical trial–based cohorts to increase generalizability, were a nonsingleton birth, had an address with low geocoding quality, and were not born between 2005 and 2019, for a final sample of 5520 children across 20 cohort sites (eFigure 1 in Supplement 1). This cohort study followed the Strengthening the Reporting of Observational Studies in Epidemiology (STROBE) reporting guideline.

### Exposure Assessment: Prenatal Water Fluoride

Public water fluoride concentrations were assigned at the census tract level. This analysis used previously developed census tract–level estimates of public water fluoride concentrations.^16^ Estimates from more than 690 000 routine compliance monitoring records were collected by public water systems to evaluate compliance with the maximum contaminant level and reported to the US Environmental Protection Agency (EPA).^17^ This approach was previously validated while examining arsenic and uranium in 2 multisite cohorts via biomarker data.^16,18^ To reduce differential missingness and bias,^19^ census tract–level estimates were aggregated to 3-year periods corresponding to the US EPA Standardized Monitoring Framework from 2005 through 2019 (eg, 2005-2007).^16,17^ Fluoride concentration metrics are reported in both milligrams per liter and micrograms per liter. We reported water fluoride exposure using the micrograms per liter metric to better represent the precision of the range of exposure and improve communication and comparisons with other contaminants, in accordance with updated reporting conventions of other low-level exposures (eg, lead).^20^ To create prenatal, area-level, time-weighted, public water fluoride exposure estimates, census tract–level water fluoride estimates were assigned to each month of pregnancy using geocoded birthing parent residential addresses. This approach accounted for residential moves across tracts and time-varying exposures for pregnancies spanning multiple exposure periods. Detailed exposure assignment information has been previously published.^16^

### Children’s Emotional and Behavioral Problems: Child Behavior Checklist

To measure children’s emotional and behavioral problems, we used the Child Behavior Checklist (CBCL), a parent-report assessment of internalizing and externalizing problems among children.^21,22^ For participants with multiple CBCL assessments, analyses were restricted to T scores from the earliest available assessment (as it was the closest to the time of the exposure). Developmentally appropriate CBCL versions were given based on child age; either the preschool version (ages 1.5-5 years; n = 3580; mean [SD] age, 3.9 [1.1] years) or the school-aged version (ages 6-17 years; n = 1940; mean [SD] age, 9.4 [1.9] years) was administered at time of study visit. Age- and sex-standardized CBCL T scores were calculated from maternal responses, where higher scores indicate greater levels of internalizing and externalizing problems.

### Covariates

Covariates included child age at CBCL measurement, child sex at birth, conception season, birthing parent educational level (high school degree or equivalent or less, some college or Associate’s degree, Bachelor’s degree or higher), maternal age at delivery (<25, 25-29, 30-34, and ≥35 years), prenatal tobacco use (any or none), and parity (nulliparous or multiparous). Area-level covariates included census tract–level population density (population divided by land area in square miles, centered) and socioeconomic vulnerability index scores—a continuous measure of an area’s potential for hardship due to social and economic factors (eMethods in Supplement 1).^23^ These covariates (including race and ethnicity [American Indian, Alaska Native, Native Hawaiian, or Pacific Islander (combined due to small sample size); Asian; Black; Hispanic; White; multiple races or ethnicities; or other race or ethnicity (not further specified)]) were obtained through several sources, including parent-reported questionnaires,^15^ and were selected based on prior exposure literature.^24^ Race and ethnicity were collected to describe the sample as well as possible differential effects related to social and environmental exposures that are inequitably distributed across racial and ethnic groups that may influence the water fluoride–children’s socioemotional association.

### Statistical Analysis

All data management and analyses were completed using R, version 4.4.0 (R Project for Statistical Computing)^25^ between December 2024 and December 2025. We evaluated the association between area-level, time-weighted prenatal water fluoride exposure and CBCL outcomes across 4 different exposure parameterizations using generalized estimating equation models with an exchangeable correlation structure to account for clustering of children by cohort site. We evaluated associations with the following approaches: restricted cubic splines models, continuous linear models, and categorical models to evaluate exposure-response across quartiles and USPHS guideline recommendations. Specifically, we evaluated prenatal public water fluoride levels continuously using cubic splines to allow for nonlinear exposure responses. Second, we examined adjusted mean differences in children’s internalizing and externalizing T scores per 500.0-µg/L higher prenatal water fluoride exposure. Third, we examined the mean differences in T scores across quartiles of fluoride (≤52.3 µg/L [reference], >52.3-350.0 µg/L, >350.0-716.0 µg/L, and >716.0 µg/L). Fourth, we examined the mean differences for exposure levels above vs below (reference) the USPHS optimal water fluoridation guideline (700.0 µg/L). For each of the analyses, model 1 adjusted for child age, child sex, birthing parent age, and educational level. To control for prenatal differences in the environment and other exposures, model 2 further adjusted for parity, prenatal tobacco use, census tract–level population density, season of conception, and socioeconomic vulnerability index score. We did not adjust for birth parent and child race or ethnicity because this approach may obscure racial or ethnic disparities in both the exposure and outcome, effectively perpetuating them.^26^ Therefore, rather than adjusting for these variables, we assessed potential differential associations by race or ethnicity (effect measure modifier; eMethods in Supplement 1). For all models, to impute for missing covariates, we used multiple imputation by chained equations (5 iterations and 10 imputed datasets; eMethods in Supplement 1). Following best current practices, when interpreting effect estimates, we evaluated the direction, precision, consistency, and magnitude of effect estimates rather than relying on dichotomous significance testing.^27^

In exploratory subgroup analysis, we examined potential differential associations of prenatal public water fluoride exposure with children’s internalizing and externalizing T scores across different subgroups (to assess potential effect measure modification) using linear models that evaluated these associations per 500.0 µg/L of higher exposure to prenatal public water fluoride. We stratified by child race and ethnicity, age at outcome assessment, birthing parent educational level, and area-level socioeconomic vulnerability index. Differential associations by race and ethnicity, educational level, and socioeconomic vulnerability index score would be related to other social and environmental exposures inequitably distributed across racial and ethnic and socioeconomic subgroups or by the differential measurement error of the outcome. In addition, we stratified by child sex based on prior literature.^28^ We did not report effect estimates for smaller subgroups (ie, <50 participants). We evaluated the statistical interaction by subgroup by evaluating *P* values from Wald tests. *P* values were from 2-tailed tests and results were deemed statistically significant at *P* < .05.

In sensitivity analyses, additional models evaluated the robustness of the potential nonlinear dose-response associations using spline models by testing the sensitivity of the curve shape to different knot locations set to the 50th and 90th percentiles. Furthermore, as an additional sensitivity analysis, we used change point models to evaluate whether changes in linear associations were detected across the exposure distribution. We compared model fit across a series of models, allowing for a varying change point (slope change) at incremental fluoride concentrations (eMethods in Supplement 1). To further assess the association of the measurement error of the exposure, we restricted our main analyses to participants (1) residing in states that publish high-quality shapefiles of public water system distribution boundaries, where the measurement error of the exposure is smaller (eMethods in Supplement 1); (2) who did not move addresses during pregnancy; and (3) who noted that their primary source of water during pregnancy was public tap water. We also repeated our main analyses after removing each cohort site (leave-1-out analysis) to identify potentially influential cohorts and after further adjusting for exposure to public water arsenic levels (potential confounder), gestational age at birth (potential mediator), and birth weight-for-gestational age *z* score (potential mediator).

## Results

**Figure 1.**
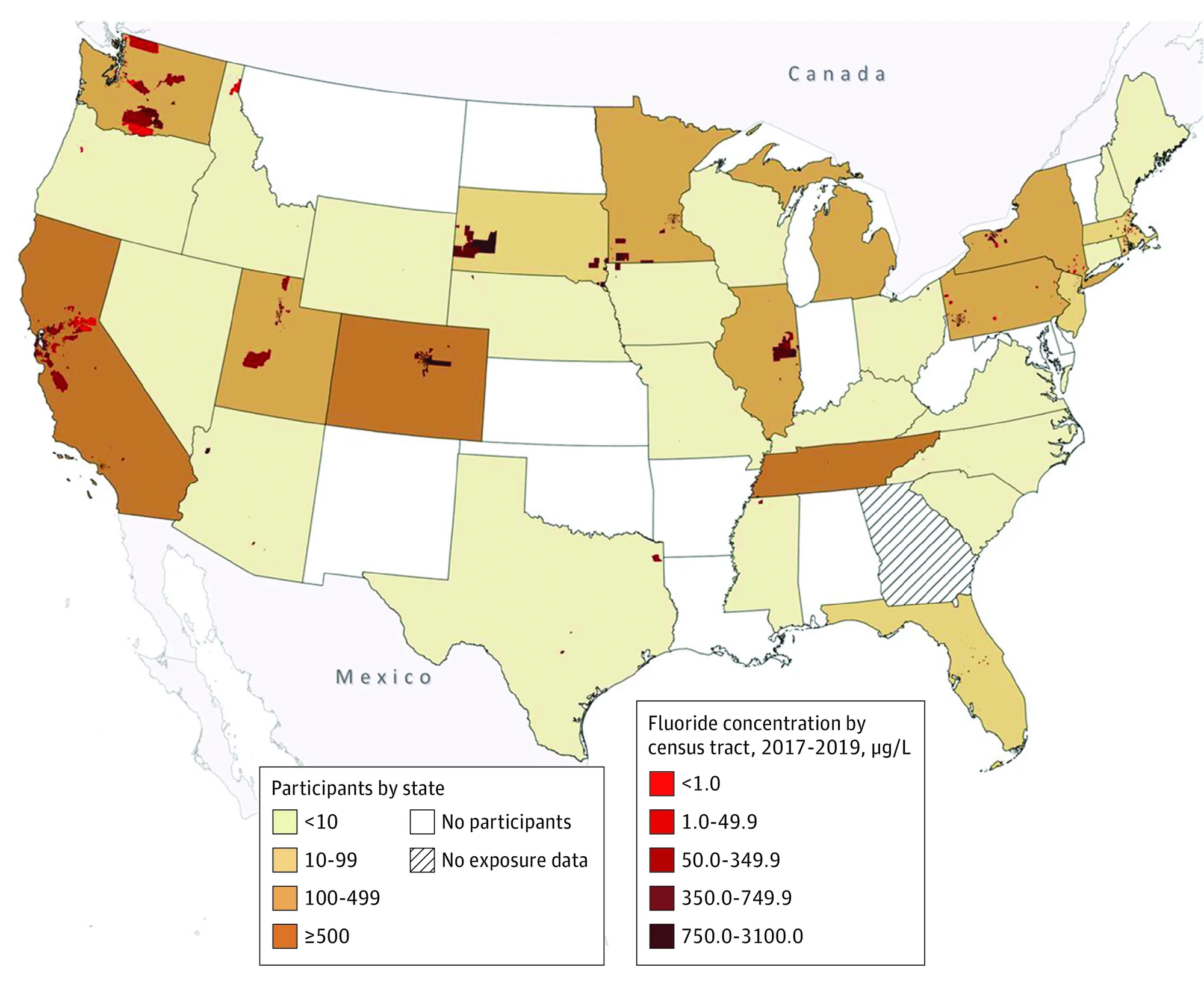
Map Showing Census Tract–Level, Population-Weighted, Mean Public Drinking Water Fluoride Concentrations (2017-2019) Across the US Overlaid With the Number of Participants From Each State (N = 5520) Individual-level, time-weighted prenatal exposure estimates were derived using birthing parent residential address, gestational timing, and census tract–level fluoride concentrations averaged across the 3-year time periods (available from 2005 to 2019). Concentration categories correspond to quartiles within our analytic sample. For fluoridated water, the US Environmental Protection Agency’s maximum contaminant level is 4000.0 µg/L, the World Health Organization Guidance Limit is 1500.0 µg/L, and the US Public Health Service optimal concentration is 700.0 µg/L.

The analysis included 5520 children (mean [SD] age, 5.8 [3.0] years; 2849 boys [51.6%] and 2671 girls [48.4%]; 57 American Indian, Alaska Native, Native Hawaiian, or Pacific Islander [1.0%], 340 Asian [6.1%], 839 Black [15.2%], 1371 Hispanic [24.8%], 3337 White [60.5%], 634 multiple races or ethnicities [11.5%], and 179 other race or ethnicity [3.2%]) (Table 1). At birth, participants resided in 2604 tracts, within 208 counties and 34 states (Figure 1). Exposure to prenatal public water fluoride concentrations ranged from less than 0.1 to 3272.5 µg/L (mean [SD] concentration, 407.9 [361.0] µg/L [0.4 (0.4) mg/L]; range, <1.4-3272.5 µg/L [<0.001-3.3 mg/L]) (Table 1). A total of 1522 participants (27.6%) had exposure to prenatal water fluoride above the USPHS optimal water fluoridation guideline (>700.0 µg/L), while 3998 (72.4%) had exposure at or below this level. See Table 1 for additional descriptive data of the study sample.

**Table 1.** Participant Characteristics Overall and Stratified by Prenatal Public Drinking Water Fluoride Quartiles

Participant characteristic	Water fluoride level, µg/L^a^				
	Overall (N = 5520)	Quartile 1, ≤52.3 (n = 1429)	Quartile 2, >52.3-350.0 (n = 1336)	Quartile 3, >350.0-716.0 (n = 1474)	Quartile 4, >716.0 (n = 1281)
Maternal age at birth, mean (SD), y	30.4 (5.4)	30 (5.7)	30.3 (5.2)	30.9 (5.4)	30.5 (5.5)
Missing, No. (%)	2 (0.04)	0	1 (0.1)	1 (0.1)	0
Maternal age categories, No. (%)					
<25 y	947 (17.2)	295 (20.6)	211 (15.8)	224 (15.2)	217 (16.9)
25-29 y	1499 (27.5)	388 (27.2)	415 (31.1)	367 (24.9)	329 (25.7)
30-34 y	1916 (34.7)	468 (32.8)	445 (33.3)	539 (36.6)	464 (36.2)
≥35 y	1156 (20.9)	278 (19.2)	264 (19.8)	343 (23.3)	271 (21.2)
Missing	2 (0.04)	0	1 (0.1)	1 (0.1)	0
Child race, No. (%)					
American Indian, Alaska Native, Native Hawaiian, or Pacific Islander^b^	57 (1.0)	12 (0.8)	16 (1.2)	19 (1.3)	10 (0.8)
Asian	340 (6.1)	116 (8.1)	82 (6.1)	88 (6.0)	54 (4.2)
Black	839 (15.2)	445 (31.1)	152 (11.4)	102 (6.9)	140 (10.9)
White	3337 (60.5)	666 (46.6)	845 (63.2)	981 (66.6)	841 (65.7)
Multiple races	634 (11.5)	139 (9.7)	163 (12.2)	171 (11.6)	161 (11.6)
Other^c^	179 (3.2)	28 (2.0)	28 (2.1)	67 (4.5)	56 (4.4)
Missing	134 (2.4)	23 (1.6)	50 (3.7)	46 (3.1)	15 (1.2)
Child ethnicity, No. (%)					
Hispanic	1371 (24.8)	246 (17.2)	320 (24.0)	506 (34.3)	299 (23.3)
Non-Hispanic	4130 (74.8)	1173 (82.1)	1012 (75.7)	964 (65.4)	981 (76.6)
Missing	19 (0.3)	10 (0.7)	4 (0.3)	4 (0.3)	1 (0.1)
Maternal educational level, No. (%)					
≤High school degree or equivalent	658 (11.9)	156 (10.9)	108 (8.1)	252 (17.1)	142 (11.1)
Some college or Associate’s degree	1410 (25.5)	368 (25.8)	373 (27.9)	373 (25.3)	296 (23.1)
≥Bachelor’s degree	3445 (62.4)	903 (63.2)	853 (63.8)	847 (57.5)	842 (65.7)
Missing	7 (0.1)	2 (0.1)	2 (0.1)	2 (0.1)	1 (0.1)
Child sex, No. (%)					
Female	2671 (48.4)	681 (47.7)	646 (48.4)	707 (48.0)	637 (49.7)
Male	2849 (51.6)	748 (52.3)	690 (51.6)	767 (52.0)	644 (50.3)
Child age at first CBCL assessment, mean (SD), y	5.8 (3.0)	7.3 (3.4)	6.0 (2.9)	4.6 (2.2)	5.4 (2.6)
Child age categories, No. (%)					
<6 y	3580 (64.9)	679 (47.5)	843 (63.1)	1237 (83.9)	821 (64.1)
≥6 y	1940 (35.1)	750 (52.5)	493 (36.9)	237 (16.1)	460 (35.9)
Parity prior to birth ≥1, No. (%)					
Multiparous	2931 (53.1)	771 (54.0)	766 (57.3)	827 (56.1)	567 (44.3)
Nulliparous	2100 (38.0)	548 (38.3)	493 (36.9)	571 (38.7)	488 (38.1)
Missing	489 (8.9)	110 (7.7)	77 (5.8)	76 (5.2)	226 (17.6)
Prenatal tobacco use, No. (%)					
No	4960 (89.9)	1321 (92.4)	1204 (90.1)	1290 (87.5)	1145 (89.4)
Yes	289 (5.2)	75 (5.2)	63 (4.7)	65 (4.4)	86 (6.7)
Missing	271 (4.9)	33 (2.3)	69 (5.2)	119 (8.1)	50 (3.9)
Socioeconomic vulnerability index score, mean (SD)^d^	0.49 (0.29)	0.50 (0.29)	0.46 (0.27)	0.53 (0.29)	0.47 (0.31)
Emotional and behavioral problem outcomes					
Internalizing T scores, mean (SD)	45.5 (10.6)	45.7 (10.6)	45.6 (10.5)	44.6 (10.6)	46.1 (10.5)
Scores above clinical range, No. (%)	619 (11.2)	NA	NA	NA	NA
Externalizing T scores, mean (SD)	44.8 (10.3)	45.1 (10.0)	44.7 (10.6)	44 (10.1)	45.4 (10.4)
Scores above clinical range, No. (%)	478 (8.7)	NA	NA	NA	NA

Abbreviations: CBCL, Child Behavior Checklist; NA, not applicable.

^a^ Prenatal public drinking water fluoride concentrations were assigned using birthing-parent–reported residential address histories during pregnancy. Exposure estimates represent individual, time-weighted, prenatal averages based on these addresses and census tract–level average community water system fluoride concentrations.

^b^ American Indian, Alaska Native, Native Hawaiian, or Pacific Islander group was combined due to small sample size.

^c^ Not further specified.

^d^ Centers for Disease Control and Prevention/Agency for Toxic Substances and Disease Registry’s socioeconomic vulnerability score (range, 0-1; 1 = most vulnerable). Assigned at the census tract level via birthing parent prenatal residential address.

**Figure 2.**
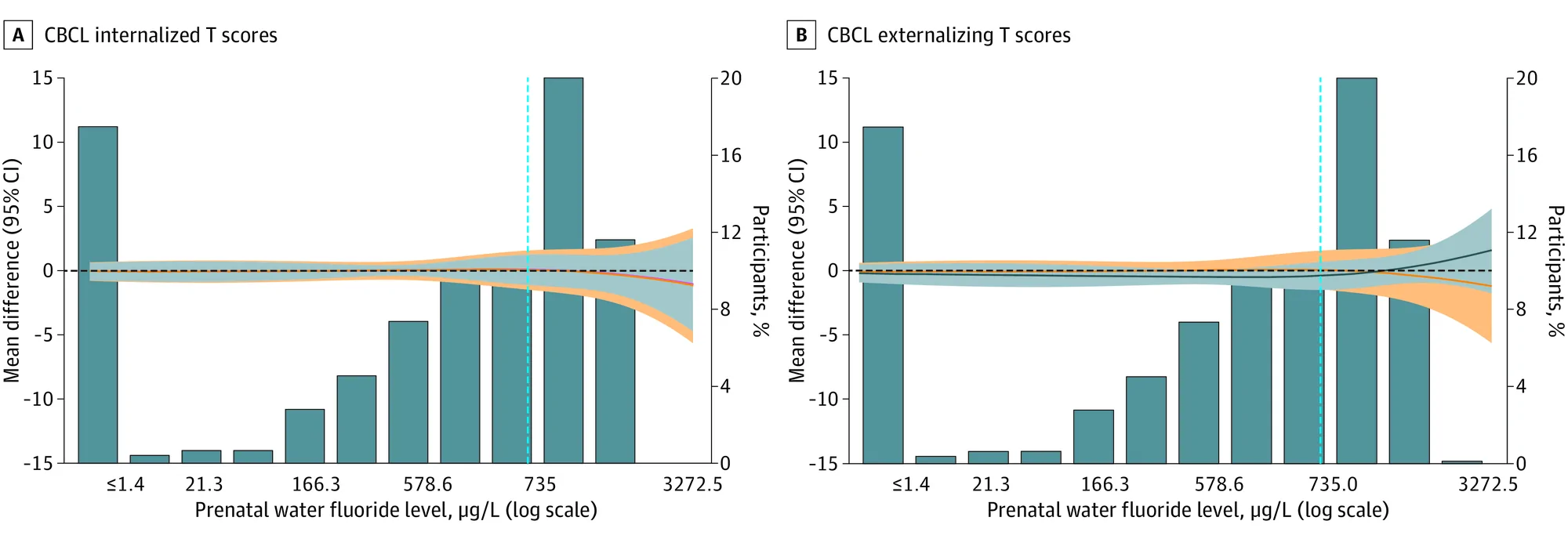
Bar Graphs Showing Restricted Cubic Spline Models of the Association Between Prenatal Public Drinking Water Fluoride Exposure and Child Behavior Checklist (CBCL) T Scores in the Environmental Influences on Child Health Outcomes (ECHO) Cohort (N = 5520)

In adjusted flexible cubic spline models, exposure to prenatal public water fluoride was not associated with children’s internalizing or externalizing problems (Figure 2). The adjusted mean difference in T scores per 500.0-µg/L higher exposure to prenatal public water fluoride was −0.15 (95% CI, −0.85 to 0.56) for internalizing problems and 0.06 (95% CI, −0.44 to 0.55) for externalizing problems, consistently showing no associations at the continuous level (Table 2). In categorical analyses comparing mean differences in T scores across quartiles of prenatal fluoride exposure, the mean difference was 0.09 (95% CI, −1.30 to 1.49) for internalizing problems and 0.14 (95% CI, −0.94 to 1.22) for externalizing problems for participants in the highest quartile of prenatal fluoride exposure (>716.0 µg/L). When comparing fluoride above vs below (reference) the USPHS optimal fluoridation guideline of 700.0 µg/L, the mean difference in T scores was 0.33 (95% CI, −0.66 to 1.32) for internalizing problems and 0.53 (95% CI, −0.35 to 1.42) for externalizing problems.

**Table 2.** Adjusted Mean Difference in CBCL T Scores Per Higher Prenatal Public Water Fluoride Exposure in the ECHO Cohort^a^

T score model^b^	Adjusted mean difference in T scores (95% CI) by water fluoride quartile				*P* value for trend^c^	Adjusted mean difference in T scores (95% CI) per increase in water fluoride level		
	Quartile 1 (≤52.3 µg/L)	Quartile 2 (>52.3-350.0 µg/L)	Quartile 3 (>350.0-716.0 µg/L)	Quartile 4 (>716.01 µg/L)		500.0 µg/L	≤700.0 µg/L	>700.0 µg/L
**Internalizing T scores**								
No.	1429	1336	1474	1281		5520	3998	1522
Model 1	0 [Reference]	0.46 (−0.58 to 1.51)	−0.54 (−1.61 to 0.53)	0.17 (−1.19 to 1.53)	.56	−0.13 (−0.83 to 0.57)	0 [Reference]	0.40 (−0.59 to 1.38)
Model 2	0 [Reference]	0.46 (−0.65 to 1.57)	−0.55 (−1.65 to 0.54)	0.09 (−1.30 to 1.49)	.84	−0.15 (−0.85 to 0.56)	0 [Reference]	0.33 (−0.66 to 1.32)
**Externalizing T scores**								
No.	1429	1336	1474	1281				
Model 1	0 [Reference]	−0.06 (−0.77 to 0.64)	−0.73 (−1.62 to 0.16)	0.43 (−0.73 to 1.59)	.69	0.17 (−0.34 to 0.68)	0 [Reference]	0.70 (−0.17 to 1.58)
Model 2	0 [Reference]	−0.17 (−0.87 to 0.52)	−0.84 (−1.71 to 0.03)	0.14 (−0.94 to 1.22)	.99	0.06 (−0.44 to 0.55)	0 [Reference]	0.53 (−0.35 to 1.42)

Abbreviations: CBCL, Child Behavior Checklist; ECHO, Environmental Influences on Child Health Outcomes.

^a^ All composite scores are age corrected and standardized (normative mean [SD], 100 [15]). Models are generalized estimating equations with participants clustered within cohort sites (n = 20) using an exchangeable correlation structure. Missingness for all covariates was imputed via multiple imputation with chained equations (via the mice package in R; 5 iterations and 10 imputed datasets).

^b^ Model 1: adjusted for birthing parent educational level (≤high school degree or equivalent [reference]; some college or Associate’s degree; or ≥Bachelor’s degree or higher) and age at delivery (<25 [reference]; 25-29; 30-34; and ≥35 years), child age at first CBCL assessment, and child sex at birth. Model 2: further adjusted for parity (dichotomous), prenatal tobacco use (dichotomous), census tract–level population density (centered), season of conception, and social vulnerability index (continuous).

^c^ *P* value trends evaluate the linear association across numeric quartile number, modeled as an ordinal variable coded 1 to 4.

### Sensitivity Analyses

After examining the sensitivity models, we found that the results remained consistent across alternative model adjustments (prenatal public water arsenic exposure, gestational age, and birth weight) and restriction criteria (nonmovers, participants who reported public drinking water as their primary water source during pregnancy, and participants with the highest-quality exposure assessments) (eTable 1 and eTable 2 in Supplement 1). Results were consistent for the alternative spline model and the leave-1-out analyses (eFigure 2 and eFigure 4 in Supplement 1). Last, linear change point models identified slope-change values at 625.0 µg/L for externalizing problems and 1825.0 µg/L for internalizing problems (eFigure 3 in Supplement 1), defined as the fluoride exposure value at which the residual sum of squares was minimized. Results from the change point models indicated an adjusted mean difference of 1.59 (95% CI, 0.72-2.47) per 500.0-µg/L increase above the inflection point for externalizing problems. Given the inflection point (1825.0 µg/L) in the change point model for internalizing T scores, the postinflection effect estimate was not interpreted due to the small sample size beyond this point (n < 10).

### Stratified Analyses

In stratified tests, despite a nonsignificant interaction term by race and ethnicity (*P* = .05; eFigure 5 in Supplement 1), analyses indicated a negative association for internalizing problems within the multiracial subgroup (mean difference in T scores, −1.34 [95% CI, –2.57 to −0.12]), whereas no such association was found for White children (mean difference in T scores, 0.22 [95% CI, –0.39 to 0.82]) or Black children (mean difference in T scores, −0.03 [95% CI, –0.88 to 0.80]). In addition, despite a nonsignificant interaction term by age (*P* = .19), stratified analyses indicated a positive association for externalizing problems within the older subgroup (≥6 years: mean difference, 0.68 [95% CI, 0.06-1.29]) whereas no such association was found for children younger than 6 years (mean difference, −0.04 [95% CI, –0.68 to 0.60]).

## Discussion

In this cohort study of 5520 children from 20 ECHO Cohort sites across the US, prenatal public water fluoride levels were not associated with children’s internalizing and externalizing problems. Across several analytic approaches, we did not observe significant associations of public drinking water fluoride exposure during pregnancy with children’s internalizing and externalizing problems However, change point sensitivity models found that higher fluoride exposure levels beyond 625.0 µg/L were associated with increases in externalizing problems. This finding is in line with existing literature,^7^ suggesting that associations with externalizing problems may emerge at higher fluoride levels. Given the high change point (1825.0 µg/L) identified in the model for internalizing T scores, the postinflection effect estimate was not interpreted due to the small sample size beyond this point (n < 10). We did not observe differences in children’s internalizing and externalizing problems when examining public drinking water fluoride levels below and above the USPHS recommended level (700.0 µg/L). Therefore, at these observed concentrations across the sample, we did not find associations of prenatal fluoride exposure with children’s socioemotional problems, and the results were consistent across all analyses. These results contrast with previous studies that have observed differences using biospecimens (eg, urine samples)^4,8^ or state-level measures of public water fluoride exposure.^7^ However, the present study used area-level water fluoride exposure at a different spatial resolution (census tract vs state) compared with previous area-level studies examining water fluoride exposure and children’s socioemotional development.

The present study helps fill important gaps regarding whether current drinking water fluoride levels and regulations at the prenatal stage pose a potential risk for children’s socioemotional problems. The mean level of water fluoride exposure in our study (407.9 µg/L) was below the recommended USPHS guideline (700.0 µg/L). Moreover, most participants (72.4%) were at or under the recommended USPHS guideline. Studying associations at these lower doses is important because they reflect the prenatal exposure levels in the US via public water systems. Furthermore, it is important to examine lower water fluoride levels to ensure the safety of current US guidelines for relatively underexamined outcomes such as socioemotional problems. The results are also policy relevant as the water fluoride was assessed using US public drinking water compliance monitoring records. When interpreting results from this study for possible policy regulations, public health policy should consider water fluoride levels within the context of all sources of fluoride exposure. Last, findings were robust to sensitivity analyses that evaluated potential sources of measurement error. Results were consistent, indicating no associations between prenatal water fluoride exposure and children’s internalizing and externalizing problems. However, small positive associations were observed for externalizing problems in change point detection models and in some subgroups, emphasizing the need for future multimethod investigations.

### Limitations

Our study has several limitations. We could not account for either postnatal water fluoride levels or other sources of fluoride exposure, such as dental care products, other beverages (ie, tea), and food. Unlike prior studies that have identified associations using biomarkers, our study is limited because we evaluated only 1 source rather than all potential sources.^4^ Although water fluoride concentration can serve as an indicator of exposure, it represents a residence-based proxy and may not capture individual-level intake or total fluoride exposure. Despite finding the same results when limiting to participants who noted that their primary source of water during pregnancy was public tap water (eTable 1 in Supplement 1), the exact water drinking behavior was not recorded. In addition, we could not account for secondary water fluoride exposure occurring in other places (eg, school, work, or place of worship). Therefore, effect estimates may be impacted by measurement error, especially for participants who reside in states that do not have high-quality assessments of the water system service area. These differential errors result from some states publishing high-quality service area shapefiles, while other service area boundaries are based solely on boundaries modeled by the US EPA.^29^ In addition, water level estimates at the area level may be underestimating an individual’s total fluoride intake, and exposure misclassification may reduce the power to detect an association. Last, our sample of participants was predominantly White, non-Hispanic, and college educated. Because of these limitations, future research is needed to further examine and assess public drinking water in psychological and epidemiologic studies.

## Conclusions

In this cohort study of children across the US, we did not observe associations between prenatal fluoride exposure via public water fluoride and children’s internalizing and externalizing problem T scores. These findings highlight that prenatal water fluoride levels near or below the current USPHS guideline (700.0 µg/L) were not associated with children’s emotional and behavioral problems. Some positive associations were observed at higher levels only after using change point models for externalizing problems and for some subgroups. Additional studies are needed to further investigate this association, especially examining other sources of fluoride that may be associated with children’s socioemotional problems and other proximal developmental outcomes.

## Notes

Supplement 1. **eFigure 1. **Exclusion Criteria **eMethods. **Detailed Description of Exposure Assessment and Statistical Analysis **eTable 1.** Sensitivity Analyses Evaluating the Association Between a 500 µg/L Higher Prenatal Public Water Fluoride Exposure and Per Log-Doubling of the Exposure and CBCL Scores in the ECHO Cohort Consortium, Considering Alternative Restriction Criteria and Model Adjustments **eTable 2.** Sensitivity Analyses Evaluating the Association Between Quartile Prenatal Public Water Fluoride Exposure Child Internalizing and Externalizing T Scores in the ECHO Cohort Consortium, Considering Alternative Restriction Criteria and Model Adjustments **eFigure 2. **Restricted Cubic Spline Models of the Association Between Prenatal Public Drinking Water Fluoride Exposure and CBCL Scores in the ECHO Cohort (N=5,520), With Full Distribution of Exposure Depicted **eFigure 3. **Sensitivity Analyses Evaluating the Association Between Prenatal Public Water Fluoride Exposure and CBCL Scores in the ECHO Cohort Consortium Using a Change-Point Model **eFigure 4.** Results of Sensitivity Analysis Removing Each Individual Cohort (“Leave-One-Out”) to Identify Influential Cohorts in the ECHO Cohort **eFigure 5. **Associations Between Prenatal Public Water Fluoride and the Child Behavioral Checklist Composite Scores in the ECHO Cohort in Stratified Subgroup Analyses
eReferences.

Supplement 2. **Noauthor Collaborators.** Environmental Influences on Child Health Outcomes (ECHO) Cohort Consortium Members

Supplement 3.
Data Sharing Statement
