# Supplementary material for: Prenatal Water Fluoride Exposure and Children’s Behavioral Problems
Source: JAMA Netw Open. 2026 Sep 3;9(9):e2629389. doi: 10.1001/jamanetworkopen.2026.29389 (PMC13543027; doi:10.1001/jamanetworkopen.2026.29389)
Supplement: Supplementary file 1 [file jamanetwopen-e2629389-s001.pdf]

Supplementary Online Content

Hernandez A, Nigra AE, Simon K, et al; Environmental Influences on Child Health Outcomes (ECHO) Cohort Consortium. Prenatal water fluoride exposure and children's behavioral problems. *JAMA Netw Open.* 2026;9(8):e2629389.
doi:10.1001/jamanetworkopen.2026.29389

eFigure 1. Exclusion Criteria
eMethods. Detailed Description of Exposure Assessment and Statistical Analysis
eTable 1. Sensitivity Analyses Evaluating the Association Between a 500 µg/L Higher Prenatal Public Water Fluoride Exposure and Per Log-Doubling of the Exposure and CBCL Scores in the ECHO Cohort Consortium, Considering Alternative Restriction Criteria and Model Adjustments
eTable 2. Sensitivity Analyses Evaluating the Association Between Quartile Prenatal Public Water Fluoride Exposure Child Internalizing and Externalizing T Scores in the ECHO Cohort Consortium, Considering Alternative Restriction Criteria and Model Adjustments
eFigure 2. Restricted Cubic Spline Models of the Association Between Prenatal Public Drinking Water Fluoride Exposure and CBCL Scores in the ECHO Cohort (n= 5,520), With Full Distribution of Exposure Depicted
eFigure 3. Sensitivity Analyses Evaluating the Association Between Prenatal Public Water Fluoride Exposure and CBCL Scores in the ECHO Cohort Consortium Using a Change-Point Model
eFigure 4. Results of Sensitivity Analysis Removing Each Individual Cohort ("Leave-One-Out") to Identify Influential Cohorts in the ECHO Cohort
eFigure 5. Associations Between Prenatal Public Water Fluoride and the Child Behavioral Checklist Composite Scores in the ECHO Cohort in Stratified Subgroup Analyses
eReferences.

This supplementary material has been provided by the authors to give readers additional information about their work.

© 2026 Hernandez A et al. *JAMA Network Open.*

eFigure 1. Exclusion Criteria

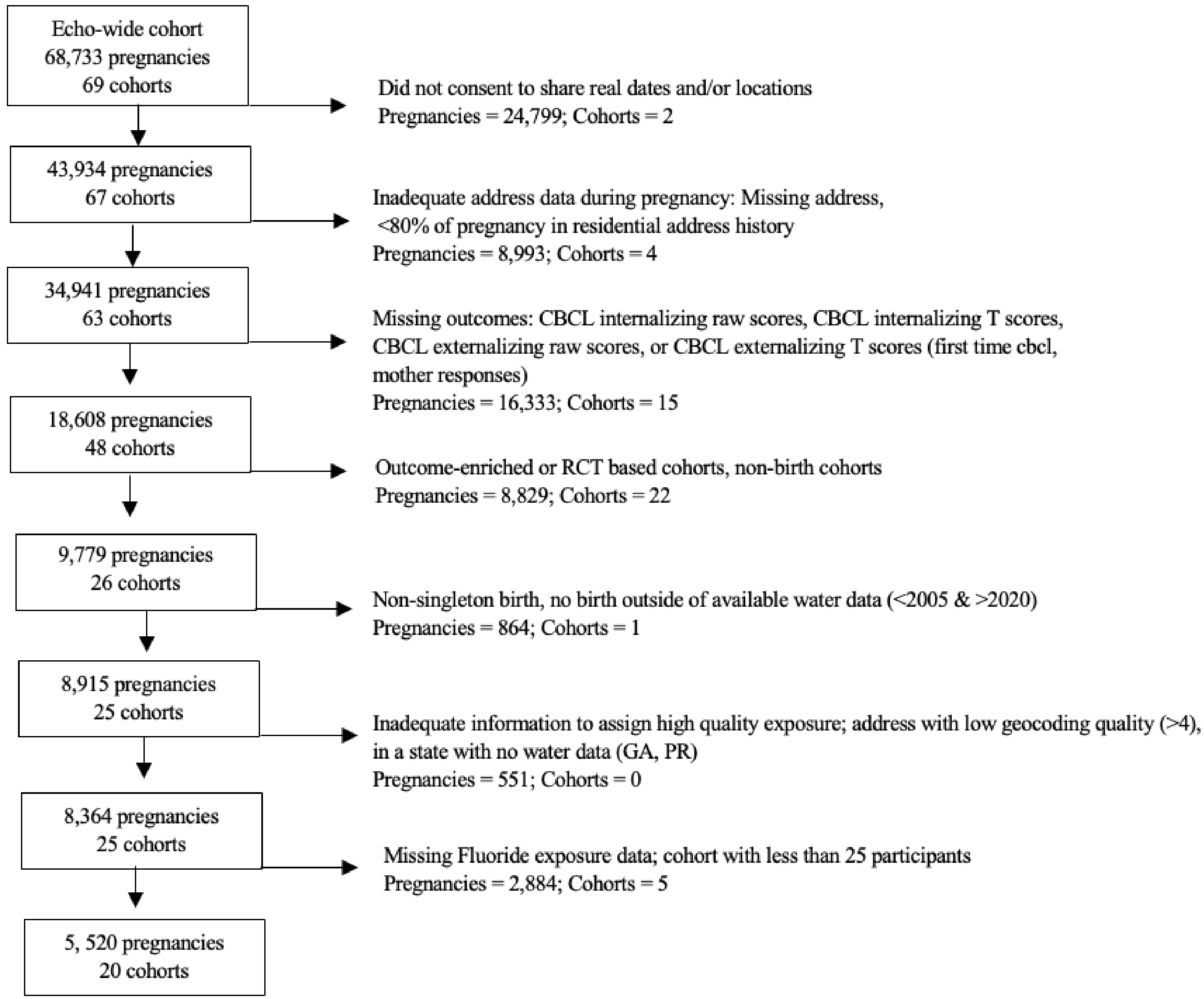

© 2026 Hernandez A et al. *JAMA Network Open.*

eMethods. Detailed Description of Exposure Assessment and Statistical Analysis

Tract-level public water fluoride concentrations.

Detailed methods describing the development of tract-level public water fluoride concentrations have been previously published.[1,2] Broadly, we assigned previously developed tract-level estimates of average, population-weighted public water fluoride concentrations to pregnancy residential address histories. This method was previously validated for water arsenic estimates in 8,000 participants from two NIH-funded, multi-site cohorts (which included both urban and rural areas) with urinary biomarkers reflecting total internal dose.[1,3] Tract-level estimates were generated by aggregating community water system fluoride concentrations to 2010 tract boundaries and applying population weights. The previously generated community water system fluoride concentrations were developed from >690,000 routine compliance monitoring records collected by >37,000 community water systems, which were collected by the U.S. Environmental Protection Agency (USEPA) for the Contaminant Occurrence Database supporting the Third (covering years 2006-2011) and Fourth (covering years 2012-2019) Six Year Review. The USEPA's Third and Fourth Six Year Review databases are the most recently available nationwide dataset of routine compliance monitoring records, representing >95% of all public water systems nationwide, and are the most extensive database on public drinking water contaminant occurrence that the USEPA has ever collected.[4–6] The USEPA conducted extensive quality control and data cleaning prior to publishing the Six Year Review databases.[4,5,7] In order to reduce differential missingness and bias,[3] community water system and tract-level estimates were aggregated to three-year time periods corresponding with the USEPA's Standardized Monitoring Framework[7] time periods (e.g., 2014-2016, 2017-2019).[6,8,9] Water system level concentrations reflect concentrations distributed to consumers in finished water (i.e., after potential treatment at the utility).[6]

Community water systems can serve multiple tracts and tracts can be served by multiple community water systems. We leveraged nationwide community water system service boundaries modeled and published by the USEPA.[10,11] Water systems were assigned to tracts by intersecting tract boundaries with water system service boundaries.[7–9] Compliance monitoring records reported different analytical limits of detection. From 2006 through 2019, the lowest method detection limits for fluoride ranged from 2.0 to >0.1 µg/L. Before aggregating to the tract-level, we imputed water system fluoride values less than the record specific detection limit as the detection limit divided by the square root of two. As such, tract-level estimates less than or equal to 1.4 µg/L reflect either

© 2026 Hernandez A et al. *JAMA Network Open.*

(a) fluoride concentrations measured below the limit of detection, or (b) very low fluoride concentrations measured above the detection limit at laboratories with unusually high precision and low detection limits. Tract-level estimates were aggregated to three-year time periods corresponding with the USEPA's Standardized Monitoring Framework[12] time periods (e.g., 2014-2016, 2017-2019), reflecting USEPA compliance monitoring periods.[13] Tract-level arsenic concentrations (used as a covariate in sensitivity analyses) were developed following this same approach.[1,2] To develop individual-level, time-weighted, average prenatal public water fluoride concentrations, each pregnancy month was assigned the corresponding tract-level fluoride average. We anticipate there may be some differential measurement error in the exposure by rural vs urban status and potentially by birthing parent race and ethnicity because of differential measurement error in estimated USEPA community water system boundaries estimates. Such differential error results from some states publishing high-quality service area shapefiles, while other service area boundaries are solely based on boundaries modeled by the USEPA.[10]

Covariates

Individual-level covariates included child sex (female / male [ref]), child age during the first time parents filled out the Child Behavioral Checklist (CBCL; 1-17 years of age), conception season (categorical: spring [March, April, May] / summer [June, July, August] [ref] / fall [September, October, November] / winter [December, January, February]), birthing parent education (≤ high school degree or equivalent / some college or Associate's degree / Bachelor's degree or higher [ref]), age at delivery (<25 [ref] / 25-29 / 30-34 / ≥35), prenatal tobacco use (any / none [ref]), parity (nulliparous [ref] / multiparous), gestational age at birth (in sensitivity analyses), and birthweight-for-gestational age z-scores (sensitivity analyses). Area-level covariates included tract-level population density (population divided by land area in square miles, centered), tract socioeconomic vulnerability index score (continuous, higher scores indicate higher socioeconomic vulnerability), and prenatal public water arsenic estimates (continuous, μg/L, in sensitivity analyses). The socioeconomic vulnerability index score was developed by the Centers for Disease Control and Prevention/Agency for Toxic Substances and Disease Registry (CDC/ATSDR) and derived from US Census American Community Survey estimates of median household income and the percent of adults who are unemployed, living below the poverty line, and without a high school diploma.[14] Data from the 2010, 2014, 2016, 2018, and 2020 datasets were utilized in this analysis.

Statistical analysis

© 2026 Hernandez A et al. *JAMA Network Open.*

For all models, we used multiple imputation by chained equations (MICE, n=5 iterations and 10 imputed datasets) to impute missing covariates. Missingness ranged from 0.3% (child ethnicity) to 4.9% (prenatal tobacco use) for individual-level covariates and from 0% (socioeconomic vulnerability index, population density) to 14.9% (arsenic, sensitivity analyses) for area-level covariates.

Sensitivity analyses

Change-point model

To further evaluate the point of departure along the x-axis (fluoride concentration value) associated with a decline CBCL scores, we additionally evaluated linear change-point models.[15] Change-point models are linear, two-piece spline models that incorporate a change in the slope of the association (interaction term) across a vector of predictor values (termed the candidate change-points). The optimal change-point is identified as the candidate change-point resulting in the lowest residual sum of squares (RSS). We evaluated a series of candidate change-point values ranging from 100 to 2400 µg/L in increments of 25 µg/L. This approach identified 1,825 µg/L and 625 µg/L as the optimal change-points for internalizing and externalizing composite scores, respectively (see eFigure 2 for a summary of RSS values across the candidate change-point values). All change-point models were conducted using imputed data (as previously described) and fit using generalized estimating equation (GEE) to account for clustering.

© 2026 Hernandez A et al. *JAMA Network Open.*

eTable 1. Sensitivity Analyses Evaluating the Association Between a 500 µg/L Higher Prenatal Public Water Fluoride Exposure and Per Log-Doubling of the Exposure and CBCL Scores in the ECHO Cohort Consortium, Considering Alternative Restriction Criteria and Model Adjustments. Prenatal public water fluoride exposure was assigned using maternal reported residential address history during pregnancy as individual, time-weighted concentrations from tract-level, population-weighted average concentrations. Models are generalized estimating equations (GEE) with participants clustered within cohorts (n=20) using an exchangeable correlation structure. Covariate missingness was imputed using multiple imputation with chained equations. Model 1 is adjusted for child sex at birth, child age during CBCL, birthing parent age, and education (categorical). Model 2 is further adjusted for parity (dichotomous), prenatal tobacco use (dichotomous), tract-level population density (centered), season of conception, and social vulnerability index (continuous).

	Internalizing T Scores Mean difference (95% CI)	Externalizing T Scores Mean difference (95% CI)
**Alternative model adjustments.** Analyses include all participants with available fluoride estimates and Child Behavioral Checklist Scores		
Model 2 adjustments + prenatal public water arsenic exposure	-0.15 (-0.85,0.57)	0.06 (-0.44,0.56)
n	5520	5520
Model 2 adjustments + gestational age at birth + birthweight for gestational age z-score	-0.14 (-0.84,0.57)	0.06 (-0.44,0.56)
n	5520	5520
**Alternative restriction criteria.**		
Non-movers (Model 2 adjustments)	-0.06 (-0.82,0.71)	0.23 (-0.33,0.79)
n	4596	4596
Water Source (Public Drinking Water)	0.35 (-1.1,1.81)	0.31 (-0.97,1.58)
n	1938	1938
High quality exposure assessment	-0.24 (-1.28,0.8)	-0.24 (-0.74,0.26)
n	3375	3375

	<=700 µg/L	>700 µg/L
**Internalizing T Scores**		
Water Source (Public Drinking Water)	0 (Reference)	0.58 (-1.64,2.80)
n	1493	445
**Externalizing T Scores**		
Water Source (Public Drinking Water)	0 (Reference)	0.21 (-1.77,2.19)
n	1493	445

© 2026 Hernandez A et al. *JAMA Network Open.*

eTable 2. Sensitivity Analyses Evaluating the Association Between Quartile Prenatal Public Water Fluoride Exposure Child Internalizing and Externalizing T Scores in the ECHO Cohort Consortium, Considering Alternative Restriction Criteria and Model Adjustments. Prenatal public water fluoride exposure was assigned using maternal reported residential address history during pregnancy as individual, time-weighted concentrations from tract-level, population-weighted average concentrations. Models are generalized estimating equations (GEE) with participants clustered within cohorts (n=20) using an exchangeable correlation structure. Covariate missingness was imputed using multiple imputation with chained equations. Model 1 is adjusted for child sex at birth, child age during CBCL, birthing parent age, and education (categorical). Model 2 is further adjusted for parity (dichotomous), prenatal tobacco use (dichotomous), tract-level population density (centered), season of conception, and social vulnerability index (continuous).

	**Quartile 1 ≤52.33 μg/L**	**Quartile 2 >52.33 – 350 μg/L**	**Quartile 3 >350 – 716.01 μg/L**	**Quartile 4 >716.01 μg/L**
**Internalizing T Scores**				
**Alternative model adjustments.** Analyses include all participants with available fluoride estimates and Child Behavioral Checklist Scores				
Model 2 adjustments + prenatal public water arsenic exposure	0.00 (reference)	0.46 (-0.65,1.57)	-0.55 (-1.65,0.54)	0.10 (-1.31,1.51)
Model 2 adjustments + gestational age at birth + birthweight for gestational age z-score	0.00 (reference)	0.50 (-0.58,1.58)	-0.52 (-1.61,0.57)	0.12 (-1.27,1.51)
**Alternative restriction criteria.**				
Non-movers (Model 2 adjustments)	0.00 (reference)	0.46 (-0.66,1.57)	-0.43 (-1.74,0.89)	0.28 (-1.22,1.78)
Water Source (Public Drinking Water)	0.00 (reference)	0.08 (-1.27,1.44)	-0.76 (-2.21,0.70)	0.52 (-2.2,3.24)
High quality exposure assessment	0.00 (reference)	0.55 (-1.16,2.27)	-0.6 (-1.95,0.75)	-0.14 (-2.18,1.89)
**Externalizing T Scores**				
**Alternative model adjustments.** Analyses include all participants with available fluoride estimates and Child Behavioral Checklist Scores				
Model 2 adjustments + prenatal public water arsenic exposure	0.00 (reference)	-0.17 (-0.86,0.51)	-0.84 (-1.71,0.03)	0.17 (-0.93,1.26)
Model 2 adjustments + gestational age at birth + birthweight for gestational age z-score	0.00 (reference)	-0.13 (-0.81,0.54)	-0.81 (-1.66,0.04)	0.17 (-0.92,1.25)
**Alternative restriction criteria.**				
Non-movers (Model 2 adjustments)	0.00 (reference)	-0.23 (-0.93,0.47)	-0.77 (-1.77,0.23)	0.34 (-0.86,1.54)
Water Source (Public Drinking Water)	0.00 (reference)	-0.48 (-1.22,0.26)	-0.80 (-2.16,0.55)	-0.06 (-2.28,2.17)
High quality exposure assessment	0.00 (reference)	-0.07 (-1.1,0.95)	-0.88 (-1.86,0.11)	-0.33 (-1.59,0.93)

© 2026 Hernandez A et al. *JAMA Network Open.*

eFigure 2. Restricted Cubic Spline Models of the Association Between Prenatal Public Drinking Water Fluoride Exposure and CBCL Scores in the ECHO Cohort (n= 5,520), With Full Distribution of Exposure Depicted. Models are generalized estimating equations (GEE) with participants clustered within cohorts (n=20) using an exchangeable correlation structure. Model 1 (purple, solid line) is adjusted for child sex at birth and birthing parent age (categorical) and education (categorical). Model 2 (red, dashed line) is further adjusted for parity (dichotomous), prenatal tobacco use (dichotomous), season of conception, tract-level population density, and social vulnerability index score. The reference is set to 52.32 µg/L (corresponding to the 25th percentile) with knots at the 50th and 90th percentiles of the distribution. Values below the record-specific limit of detection were imputed as the limit of detection divided by the square root of two (values ≤1.4 µg/L represent fluoride concentrations measured below the lowest method limits of detection, which varied by year).

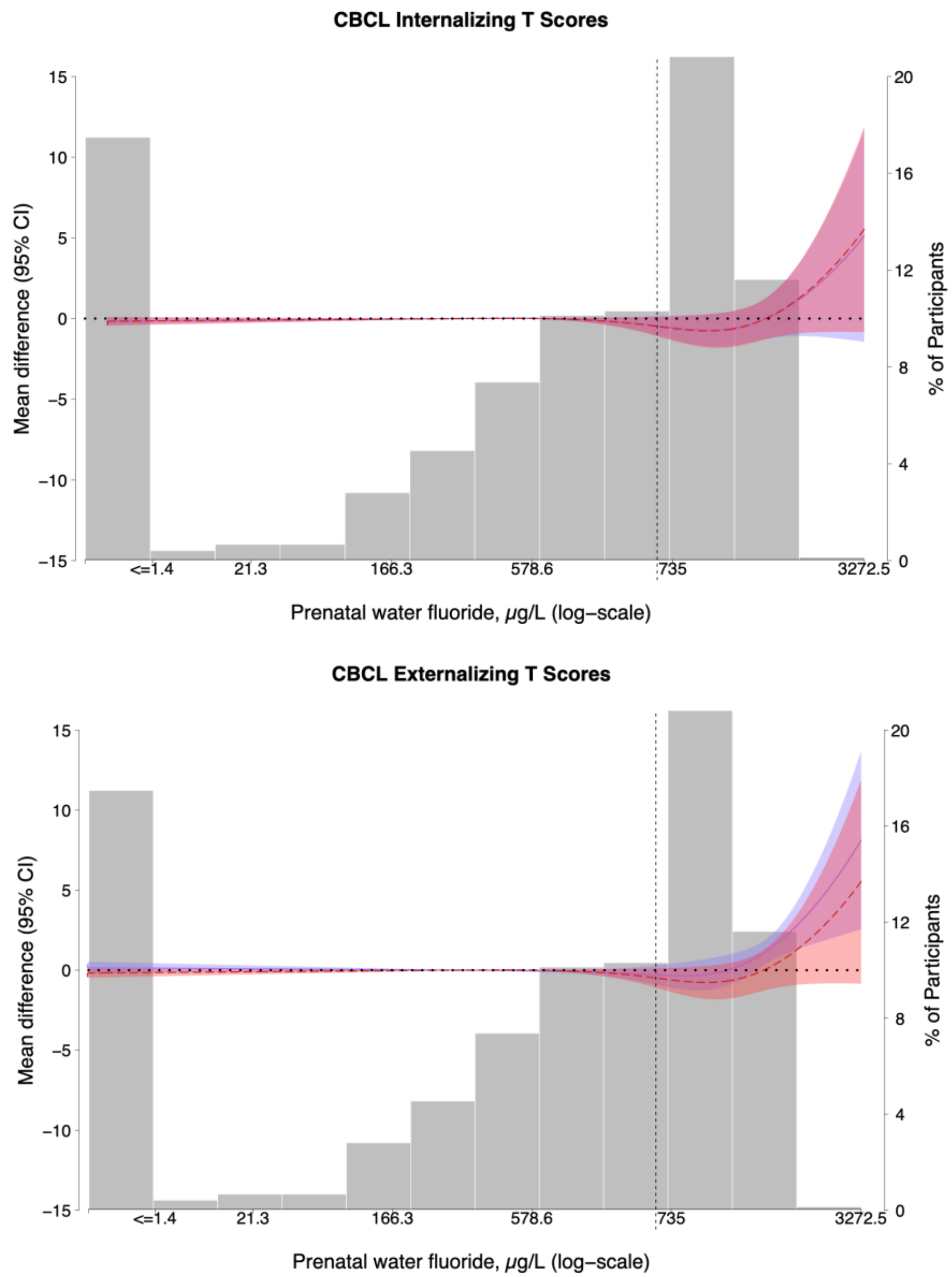

© 2026 Hernandez A et al. *JAMA Network Open.*

eFigure 3. Sensitivity Analyses Evaluating the Association Between Prenatal Public Water Fluoride Exposure and CBCL Scores in the ECHO Cohort Consortium Using a Change-Point Model. The left panels display the calculated residual sum of squares (RSS) at potential change-points for internalizing t-score (top panel) and externalizing t-scores (bottom panel). The optimal change-point is determined by the exposure value at which the RSS is smallest: 1825 µg/L for internalizing t-scores and 625 µg/L for externalizing t-scores. The right panel presents the fully adjusted mean differences and 95% confidence intervals for the association between prenatal public water fluoride exposure and internalizing and externalizing scores, separately for levels below and above the identified change-point. Mean difference values after the change-point reflect the combined sum of the slope below the change-point and the change in slope beyond the change-point.

	**Mean difference (95% CI) per 500 µg/L higher water fluoride**		
	Slope before change-point	Change in slope after change-point	Slope after change-point
Internalizing T Scores	-0.07 (-0.84, 0.70)	-4.96 (-7.2, -2.71)	-5.02 (-6.57, -3.47)
Externalizing T Scores	-0.75 (-1.55, 0.05)	2.35 (0.74, 3.95)	1.59 (0.72, 2.47)

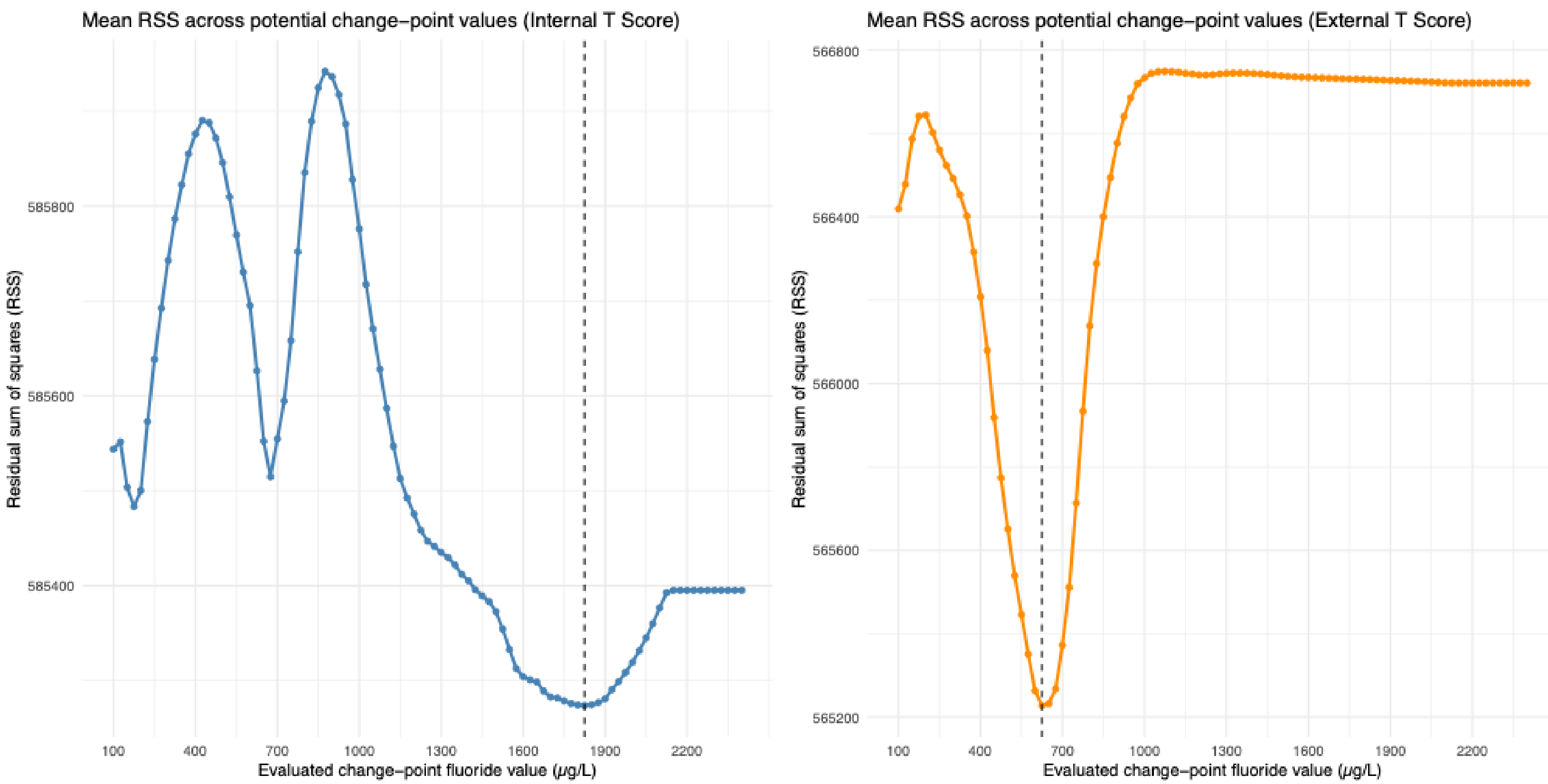

© 2026 Hernandez A et al. *JAMA Network Open.*

eFigure 4. Results of Sensitivity Analysis Removing Each Individual Cohort ("Leave-One-Out") to Identify Influential Cohorts in the ECHO Cohort. Estimates represent mean differences in scores per 500 µg/L higher prenatal public water fluoride exposure in linear models. ECHO cohort names are abbreviated, and the participant sample size is provided for each cohort. Models are adjusted for child sex at birth, child age, birthing parent education and age, parity, prenatal tobacco use, season of conception, tract-level population density, and social vulnerability index score. Socioeconomic vulnerability was assessed using the CDC/ATSDR's census tract-level socioeconomic vulnerability index.

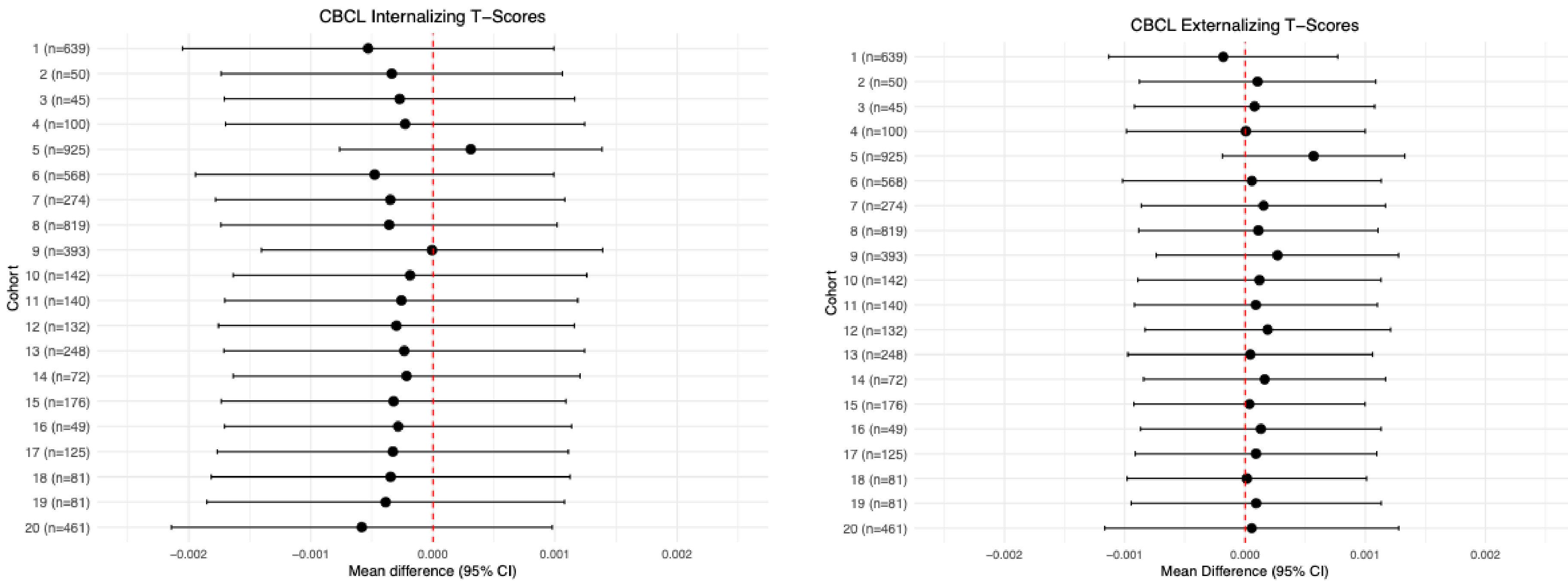

© 2026 Hernandez A et al. *JAMA Network Open.*

eFigure 5. Associations Between Prenatal Public Water Fluoride and the Child Behavioral Checklist Composite Scores in the ECHO Cohort in Stratified Subgroup Analyses. Associations represent mean differences in scores per a 500 µg/L higher prenatal public water fluoride exposure in linear models. The size of the square corresponds to the number of participants in each subgroup. We do not report results for subgroups with less than 50 participants or when models did not converge because of small sample size. Prenatal public water fluoride exposure was assigned using birthing parent residential address history during pregnancy as individual, time-weighted concentrations from residential census-tract, population-weighted average concentrations. Models are generalized estimating equations (GEE) with participants clustered within cohorts (n=20) using an exchangeable correlation structure. Missingness for all covariates was imputed via multiple imputation with chained equations (via the *mice* package in R; n=5 iterations and 10 imputed datasets). Models are adjusted for child sex at birth, child age, birthing parent education and age, parity, prenatal tobacco use, season of conception, tract-level population density, and social vulnerability index score. Socioeconomic vulnerability was assessed using the CDC/ATSDR's census tract-level socioeconomic vulnerability index. Stratified analyses and interaction terms were separately assessed across two ages of assessment (under and above 6 years of age) to evaluate whether stratified effects differed based on selected age cutoff. Child ages presented below reflect ages entered when administering the Child Behavioral Checklist. Age stratified results remained consistent when child ages were derived manually based on assessment date and child date of birth. Statistical interaction (p-interaction) was assessed using Wald tests.

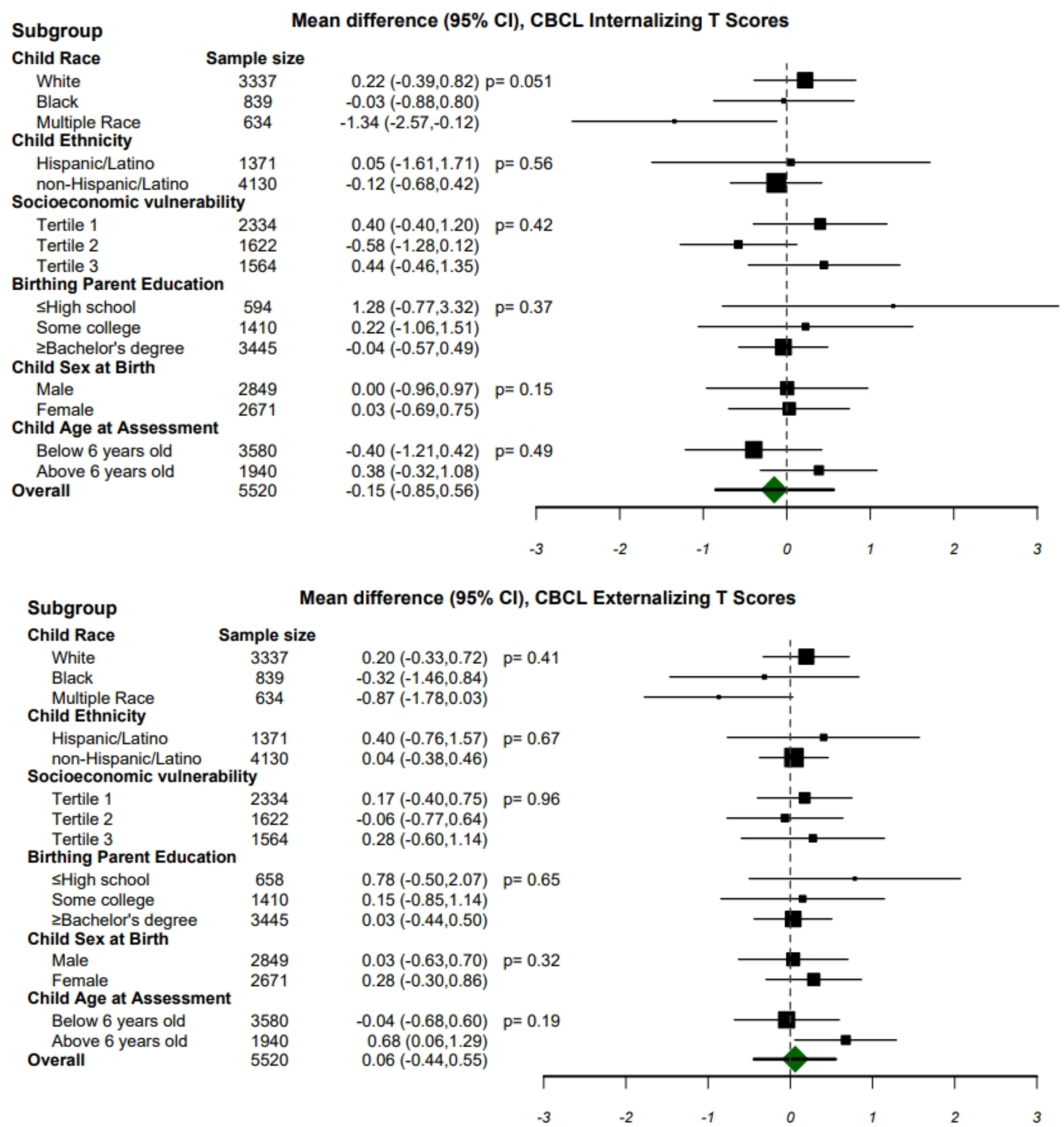

© 2026 Hernandez A et al. *JAMA Network Open.*

eReferences.

1. Bloomquist TR, Spaur M, Cerna-Turoff I, et al. Public drinking water contaminant estimates for birth cohorts in the Environmental Influences on Child Health Outcomes (ECHO) Cohort. *J Expo Sci Environ Epidemiol*. Published online August 4, 2024. doi:10.1038/s41370-024-00699-2

2. Nigra AE. Public water arsenic and birth outcomes in the Environmental Influences on Child Health Outcomes Cohort. *JAMA Netw Open*. 8(6). doi:doi:10.1001/jamanetworkopen.2025.14084.

3. Spaur M, Glabonjat RA, Schilling K, et al. Contribution of arsenic and uranium in private wells and community water systems to urinary biomarkers in US adults: The Strong Heart Study and the Multi-Ethnic Study of Atherosclerosis. *J Expo Sci Environ Epidemiol*. 2024;34(1):77-89. doi:10.1038/s41370-023-00586-2

4. U.S. Environmental Protection Agency. *Six-Year Review 3 Compliance Monitoring Data (2006-2011)*. Accessed May 15, 2025. https://www.epa.gov/dwsixyearreview/six-year-review-3-compliance-monitoring-data-2006-2011

5. U.S. Environmental Protection Agency. *The Analysis of Regulated Contaminant Occurrence Data from Public Water Systems in Support of the Third Six-Year Review of National Primary Drinking Water Regulations: Chemical Phase Rules and Radionuclides Rules.* Accessed May 15, 2025. https://www.epa.gov/sites/production/files/2016-12/documents/810r16014.pdf

6. Nigra AE, Chen Q, Chillrud SN, et al. Inequalities in Public Water Arsenic Concentrations in Counties and Community Water Systems across the United States, 2006–2011. *Environ Health Perspect*. 2020;128(12):127001. doi:10.1289/EHP7313

7. U.S. Environmental Protection Agency. *The Data Management and Quality Assurance/Quality Control Process for the Third Six-Year Review Information Collection Rule Dataset.* Accessed May 15, 2025. https://www.epa.gov/sites/default/files/2016-12/documents/810r16015_0.pdf

8. Spaur M, Bostick BC, Chillrud SN, Factor-Litvak P, Navas-Acien A, Nigra AE. Impact of lowering the US maximum contaminant level on arsenic exposure: Differences by race, region, and water arsenic in NHANES 2003–2014. *Environ Pollut*. 2023;333:122047. doi:10.1016/j.envpol.2023.122047

9. Nigra AE, Sanchez TR, Nachman KE, et al. The effect of the Environmental Protection Agency maximum contaminant level on arsenic exposure in the USA from 2003 to 2014: an analysis of the National Health and Nutrition Examination Survey (NHANES). *Lancet Public Health*. 2017;2(11):e513-e521. doi:10.1016/S2468-2667(17)30195-0

© 2026 Hernandez A et al. *JAMA Network Open.*

10. United States Environmental Protection Agency. *Community Water System Service Area Boundaries.* Accessed May 15, 2025. https://www.epa.gov/ground-water-and-drinking-water/community-water-system-service-area-boundaries

11. McDonald YJ, Anderson KM, Caballero MD, et al. A systematic review of geospatial representation of United States community water systems. *AWWA Water Sci*. 2022;4(1):e1266. doi:10.1002/aws2.1266

12. *U.S. Environmental Protection Agency. The Standardized Monitoring Framework: A Quick Reference Guide.* https://nepis.epa.gov/Exe/ZyPDF.cgi/3000667K.PDF?Dockey=3000667K.PDF

13. U.S. Environmental Protection Agency. *National Primary Drinking Water Regulations: Arsenic and Clarifications to Compliance and New Source Contaminants Monitoring.* https://www.govinfo.gov/content/pkg/FR-2001-01-22/pdf/FR-2001-01-22.pdf

14. *Centers for Disease Control and Prevention/ Agency for Toxic Substances and Disease Registry/ Geospatial Research Analysis and Services Program. CDC/ATSDR Social Vulnerability Index 2018 Database US. Accessed 16 December 2021, Https://Www.Atsdr.Cdc.Gov/Placeandhealth/Svi/Data_documentation_download.Html*.

15. Demidenko E. *Advanced Statistics with Applications in R.*; 2019.

© 2026 Hernandez A et al. *JAMA Network Open.*
