# Supplementary material for: Prenatal Water Fluoride Exposure and Children’s Behavioral Problems
Source: JAMA Netw Open. 2026 Sep 3;9(9):e2629389. doi: 10.1001/jamanetworkopen.2026.29389 (PMC13543027; doi:10.1001/jamanetworkopen.2026.29389)
Supplement: Supplementary file 3 [file jamanetwopen-e2629389-s003.pdf]

Data Sharing Statement

Hernandez. Prenatal Water Fluoride Exposure and Children's Behavioral Problems. *JAMA Netw Open*. Published August 21, 2026. doi:10.1001/jamanetworkopen.2026.29389

Data
Data available: Yes
Data types: Deidentified participant data
How to access data: Select de-identified data from the ECHO Program are available through NICHD's Data and Specimen Hub (DASH). Information on study data not available on DASH, such as some Indigenous datasets, can be found on the ECHO study DASH webpage.
When available: With publication

Supporting Documents
Document types: None

Additional Information
Who can access the data: Select de-identified data from the ECHO Program are available through NICHD's Data and Specimen Hub (DASH). Information on study data not available on DASH, such as some Indigenous datasets, can be found on the ECHO study DASH webpage.
Types of analyses: any purpose
Mechanisms of data availability: Data on the DASH platform are publicly available.
Any additional restrictions: None
